# Supplementary figures and images for: SSNIP-seq: A simple and rapid method for isolation of single-sperm nucleic acid for high-throughput sequencing
Source: PLoS One. 2022 Sep 29;17(9):e0275168. doi: 10.1371/journal.pone.0275168 (PMC9521801; doi:10.1371/journal.pone.0275168)

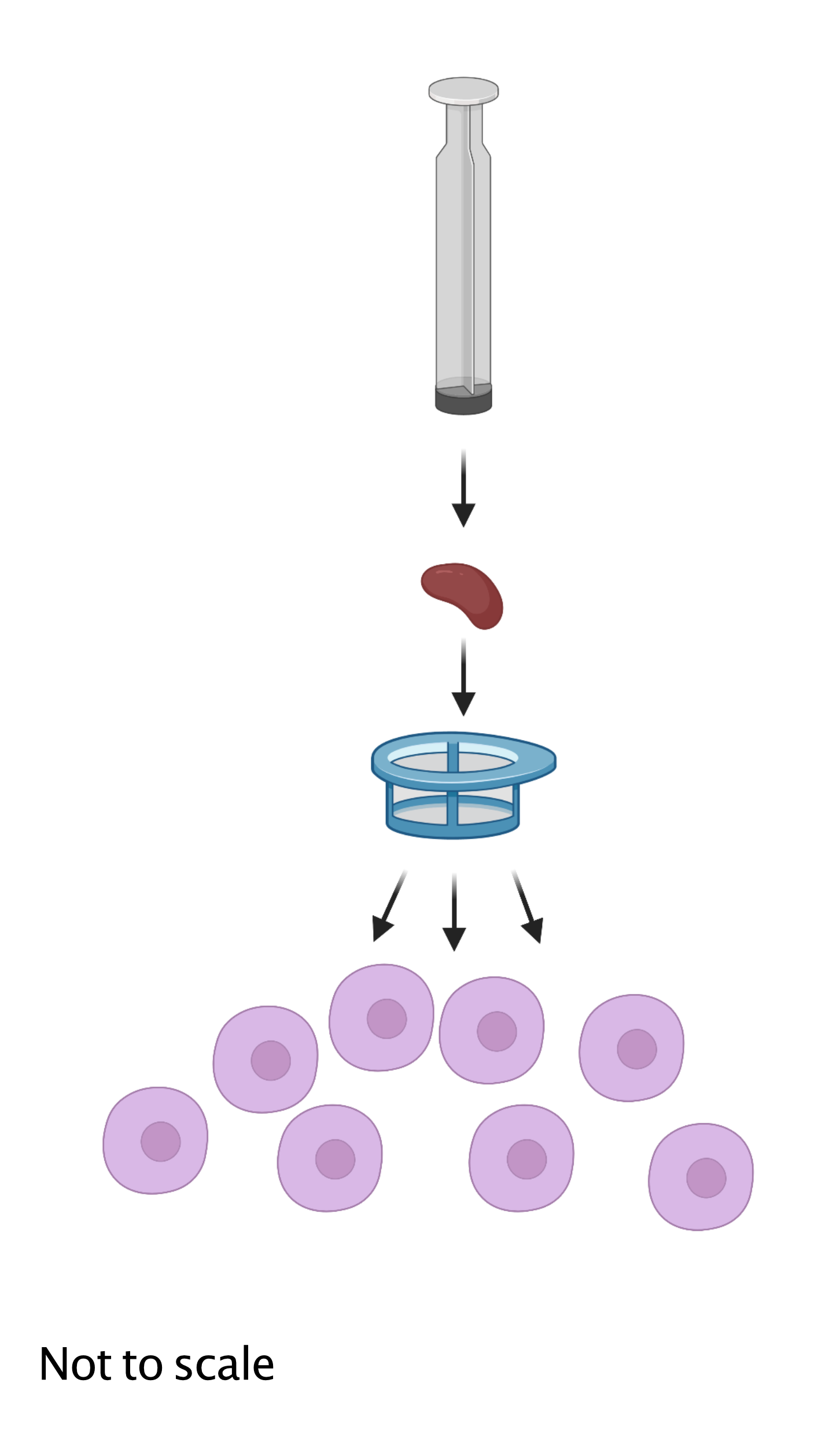

Supplement: S1 Fig — Cartoon representation of how splenic cells are obtained via homogenisation and filtration. (TIFF) [file pone.0275168.s001.tiff]
