# Supplementary material for: SSNIP-seq: A simple and rapid method for isolation of single-sperm nucleic acid for high-throughput sequencing
Source: PLoS One. 2022 Sep 29;17(9):e0275168. doi: 10.1371/journal.pone.0275168 (PMC9521801; doi:10.1371/journal.pone.0275168)
Supplement: S1 File — (PDF) [file pone.0275168.s003.pdf]

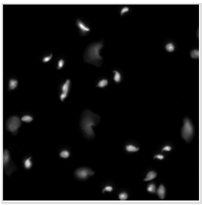

May 28, 2022

SSNIP-seq: A simple and rapid method for isolation of single-sperm nucleic acid for high-throughput sequencing

Steve Novakovic[1], Vanessa Tsui[1,2], Tim Semple[3], Luciano G Martelotto[4], Davis J Mccarthy[5,6], Wayne Crismani[1,2]

[1]The DNA Repair and Recombination Laboratory, St Vincent's Institute of Medical Research;

[2]The Faculty of Medicine, Dentistry and Health Science, The University of Melbourne;

[3]Single Cell Innovation Laboratory, Centre for Cancer Research, The University of Melbourne;

[4]University of Adelaide;

[5]Bioinformatics and Cellular Genomics, St. Vincent's Institute of Medical Research, Melbourne, Australia;

[6]Melbourne Integrative Genomics, Faculty of Science, The University of Melbourne, Melbourne, Australia

2 Works for me   Share

dx.doi.org/10.17504/protocols.io.6qpvr67jbvmk/v1

Wayne Crismani

ABSTRACT

We developed a simple and reproducible method for the isolation of haploid nuclei from fresh and frozen tests. The described protocol utilises readily available reagents in combination with flow cytometry to separate haploid and diploid nuclei. The protocol can be completed within 1 hour and the resulting individual haploid nuclei have intact morphology. The isolated nuclei are suitable for library preparation for high-throughput DNA and RNA sequencing using bulk or single nuclei. The protocol was optimised with mouse testes and we anticipate that it can be applied for the isolation of mature sperm from other mammals including humans.

DOI

dx.doi.org/10.17504/protocols.io.6qpvr67jbvmk/v1

PROTOCOL CITATION

Steve Novakovic, Vanessa Tsui, Tim Semple, Luciano G Martelotto, Davis J Mccarthy, Wayne Crismani 2022. SSNIP-seq: A simple and rapid method for isolation of single-sperm nucleic acid for high-throughput sequencing. **protocols.io** https://dx.doi.org/10.17504/protocols.io.6qpvr67jbvmk/v1

Citation: Steve Novakovic, Vanessa Tsui, Tim Semple, Luciano G Martelotto, Davis J Mccarthy, Wayne Crismani SSNIP-seq: A simple and rapid method for isolation of single-sperm nucleic acid for high-throughput sequencing https://dx.doi.org/10.17504/protocols.io.6qpvr67jbvmk/v1

This is an open access protocol distributed under the terms of the **Creative Commons Attribution License**

FUNDERS ACKNOWLEDGEMENT

Australian National Health and Medical Research Council
Grant ID: GNT1129757

Australian National Health and Medical Research Council
Grant ID: GNT1185387

LICENSE

This is an open access protocol distributed under the terms of the Creative Commons Attribution License, which permits unrestricted use, distribution, and reproduction in any medium, provided the original author and source are credited

CREATED

May 27, 2022

LAST MODIFIED

Jul 29, 2022

PROTOCOL INTEGER ID

63310

MATERIALS TEXT

Equipment and software

- Cell sorter (BD400 Class II FACSAria Fusion Cell Sorter with FACS Diva software, Becton Dickinson)
- Immunofluorescence microscope that can visualise DAPI
- Benchtop microcentrifuge with temperature control or located in a cold room. We use an Eppendorf Centrifuge 5418 in a 4 ˚C cold room.
- PCR tube adapters for the microcentrifuge
- Timer

Laboratory materials

- Flowmi cell strainers, 40 µm (Bel-Art, cat no. H13680-0040)
- DNA LoBind microcentrifuge tubes, 1.5 mL (Eppendorf, cat no. 0030 108 418)
- Round-bottom polypropylene tubes with cap, 5 mL (Falcon, cat no. 352063)
- Wide bore pipette tips, 1000 µL (Axygen, cat no. T-1005-WB-C) (optional)
- Micropipettes DV1000, DV200, DV20, D10 (THL, cat no. PZ-7901)
- Cell strainers, 70 µm (Corning, cat no. 431751)
- Tweezers with pointed tips, 115mm (Weller Erem, cat no. 5SA)
- Tissue-culture treated culture dishes, 35 x 10 mm (Corning, cat no. 430165)
- Thin wall PCR tubes with flat cap, 200 µL (Axygen, cat no. PCR-02-L-C)
- 96-well plate with round bottom (Greiner)
- Sterile syringe 0.2 µm filters, acrodisc (Pall, cat no. 2415600)
- 50 mL syringe

Citation: Steve Novakovic, Vanessa Tsui, Tim Semple, Luciano G Martelotto, Davis J Mccarthy, Wayne Crismani SSNIP-seq: A simple and rapid method for isolation of single-sperm nucleic acid for high-throughput sequencing https://dx.doi.org/10.17504/protocols.io.6qpvr67jbvmk/v1

This is an open access protocol distributed under the terms of the Creative Commons Attribution License

- 50 mL centrifuge tube
- 2 mL plastic syringe (BD, cat. no. 302204)

Biological material

One wild-type male mouse six weeks or older. This protocol was developed using C57BL/6J, FVB/N, and F1(C57BL/6J x FVB/N) mice.

Solutions

- 10x Dulbecco's Phosphate Buffered Saline (DPBS), without calcium chloride and magnesium chloride (Sigma Aldrich, cat no. D1408-500ML)
- 1x DPBS, without calcium chloride and magnesium chloride (Sigma Aldrich, cat no. D8537-500ML)
- 4, 6-Diamidino-2-phenylindole dihydrochloride (DAPI; Sigma Aldrich, cat no. D8417-1MG)
- 'Bovostar' Bovine Serum Albumin (BSA; Bovogen Biologicals, cat no. BSASAU-0.1)
- Nuclei EZ Lysis Buffer from Nuclei EZ Prep Kit (Sigma Aldrich, cat no. NUC101-1KT)
-

Buffers and Reagents

- **DAPI stock** - Prepare 1 mg / mL of DAPI in sterile water. Filtered using a 0.22 µm filter and stored at -20°C.
- **10% BSA** - Prepare a 10% BSA (w/v) stock solution with sterile water. Filter using a 0.22 µm filter and store at 4°C for up to a month.
- **Nuclei Wash Resuspension Buffer (NWRB) with 1% or 0.1% BSA (w/v)** - Prepare solution using 10x DBPS, 10% BSA stock and sterile water. Make fresh prior to use and keep on ice.
- **NWRB with 1% BSA and DAPI (10 µg / mL)** - Prepare using 10x DBPS, 10% BSA stock, DAPI stock and sterile water. Make fresh prior to use, keep on ice and protect from light.

1 **Procedure**

All steps must be completed quickly, and samples kept at 4°C at all times. The use of Lo-Bind microcentrifuge tubes is critical to avoid excessive loss of sperm cells or nuclei.

Tissue isolation

1| Sacrifice mice using CO2 or cervical dislocation following local SOPs that have been approved by an animal ethics committee.

2 2| Dissect testis and spleens from each mouse, removing as much fat and unwanted connective tissue as possible, and place each individually in a 1.5 mL microcentrifuge tube containing cold DPBS.

· If nuclei isolation is performed on the day, keep the testis on ice and begin the protocol within 30 minutes of dissection. Otherwise, the testes can be individually place in an empty 1.5 mL microcentrifuge tube and snap frozen in liquid nitrogen then stored at -80oC. We have obtained >100,000 nuclei from frozen testis samples up to 1 month after freezing. Spleen cell suspension A spleen cell suspension is formed which will serve as a diploid control.

Citation: Steve Novakovic, Vanessa Tsui, Tim Semple, Luciano G Martelotto, Davis J Mccarthy, Wayne Crismani SSNIP-seq: A simple and rapid method for isolation of single-sperm nucleic acid for high-throughput sequencing https://dx.doi.org/10.17504/protocols.io.6qpvr67jbvmk/v1

This is an open access protocol distributed under the terms of the **Creative Commons Attribution License**

3 3| Place a 70 µm cell strainer into a 50 mL centrifuge tube and wet the strainer by pipetting 1 mL cold DPBS onto the strainer mesh.

4 4| Place the whole spleen into the strainer and homogenise it using the plunger seal of a 2 mL plastic syringe. Rinse the strainer with an additional 1 mL of cold DPBS. Place the sample on ice until step 7.

· The solution should look homogenous. If the suspension contains clumps of material, it can be filtered into a second 50 mL centrifuge tube using a clean 70 µm cell strainer.

5 **Nuclei isolation**

At this stage it is anticipated that you will have:
- Either a fresh or frozen testis;
- If using a frozen testis allow it to thaw on ice for 10 minutes; and
- A spleen to serve as a diploid control

All steps are performed on ice

5| Place two 1.5 mL microcentrifuge tubes on ice and add 1 mL of chilled Nuclei EZ Lysis Buffer to each.

6 6| Transfer the fresh or thawed testis to one of the microcentrifuge tubes.

· Release the seminiferous tubules from the testis by gently squeezing the testis with a pointed tweezers until the testis burst. Gently tear and homogenise the testis and in turn the seminiferous tubules several times with the tweezers to further break them apart and allow the release of more cells into solution. The solution should be cloudy due to the high cell number.

7 7| Add 300 µL of the spleen cell suspension (from step 4) to the second microcentrifuge.

8 8| Incubate both the spleen and the testis samples on ice for 5 minutes to allow cell lysis and the release of nuclei. From here until step 18 (Cell sorting) both the testis and spleen sample are treated the same.

· After 3 minutes of the 5-minute incubation gently invert the samples two or three times to allow better mixing of the cells with the Nuclei EZ Lysis Buffer.

· For mutants with smaller testis or low sperm production it may be necessary to break the testis apart more extensively or break apart two testes in the same tube. To overcome volume constraints this may need to be performed in a 2.0 mL Lo-Bind microcentrifuge.

Citation: Steve Novakovic, Vanessa Tsui, Tim Semple, Luciano G Martelotto, Davis J Mccarthy, Wayne Crismani SSNIP-seq: A simple and rapid method for isolation of single-sperm nucleic acid for high-throughput sequencing
https://dx.doi.org/10.17504/protocols.io.6qpvr67jbvmk/v1

This is an open access protocol distributed under the terms of the **Creative Commons Attribution License**

9 9| Just prior to the end of the 5-minute incubation period, remove any large pieces of non-degraded testis debris.

· Failure to remove debris will result in unwanted clumping of debris and the target nuclei onto the bottom of the tube.

10 10| Centrifuge the samples at 500 x g for 5 minutes at 4°C. Remove the supernatant, leaving behind just enough to cover the pellet. Do not allow the pellet to dry.

11 11| Add 1 mL of Nuclei EZ Lysis Buffer. Repeat steps 8-10.

12 12| Very slowly add 1 mL of NWRB with 1% BSA and incubate the sample on ice for 5 minutes to allow buffer exchange. Take care to avoid resuspending the pellet.

13 13| After incubation gently resuspend the pellet by either inverting 5 times or pipetting up and down 5 times with a wide-bore tip.

14 14| Centrifuge at 500 x g for 5 minutes at 4°C. Remove the supernatant, leaving behind just enough to cover the pellet. Do not dry pellet.

15 15| Resuspend the pellet in 300 µL NWRB with 1% BSA and 3 µL of the DAPI stock (final concentration 10 µg / µL) by either inverting 5 times or pipetting up and down 5 times with a wide-bore tip.

· To resuspend the pellet gently invert the sample several times. If it fails to resuspend a 1 mL wide-bore pipette tip can be used to gently pipette mix the sample up and down up to 10 times
· Full resuspension of the pellet is not essential and, in our hands, still yields excess of 300,000 haploid nuclei. Excessive inverting or mixing may damage the integrity of the nuclei.

16 16| Filter the sample using a 40 µm Flowmi cell strainer into a 5 mL polypropylene tube which is kept on ice and in the dark. Cell sorting Prior to nuclei sorting turn on the cytometer as per the manufacturer's instructions. Use a collection device capable of holding 5 mL polypropylene tubes and ideally maintaining 4oC. Using diploid control material (here, the single splenic nuclei suspension) is used to identify the G1 diploid peak based on DAPI intensity). This G1 peak – of the diploid sample – is centred on the x-axis by adjusting voltage. Haploid sperm nuclei can then be distinguished from diploid nuclei using the same gating

Citation: Steve Novakovic, Vanessa Tsui, Tim Semple, Luciano G Martelotto, Davis J Mccarthy, Wayne Crismani SSNIP-seq: A simple and rapid method for isolation of single-sperm nucleic acid for high-throughput sequencing
https://dx.doi.org/10.17504/protocols.io.6qpvr67jbvmk/v1

This is an open access protocol distributed under the terms of the **Creative Commons Attribution License**

parameter but with a final DAPI intensity approximately half that of the diploid control.

17 17| Preparation of FACS collection tubes and plates – For each sample add 200 µL of NWRB with 0.1% BSA in a 5 mL polypropylene tube. For collections in 96-well plates add 50 µL of NWRB with 1% BSA.

18 18| Critical step – Set the sorter precision to '4-Way Purity', this will ensure that the final volume of the sorted nuclei is kept to a minimum (~1 nucleus sorted per nL), preventing dilution of the sample with FACS sheath solution.

· On some instruments this setting may be called 'Single Cell' precision when sorting in 96-well plates.
· It is preferable to use slow flow rates, but a medium flow rate can be used if necessary.

19 19| Using the standard workflows of your FACS facility, gate for individual cells.

20 20| Begin by visualising the DNA content of the spleen-derived nuclei sample. This serves as a diploid control, which is essential for being able to confidently identify haploid nuclei. It is therefore important to set up the gates with the DAPI signal to allow for a G1 and G2 peak of the diploid sample, as well as a haploid peak, in the same range on the x-axis. The haploid peak – on a density plot – will be half that of the diploid G1 peak.

21 21| Diploid nuclei control sorting – Sort the target number of diploid nuclei – typically 100,000 or greater – into the previously prepared 5 mL polypropylene tube or 96 well plate.

· While this sample is generally not used for library preparation and sequencing, it is helpful to perform sorting of individual nuclei from this sample as it is typically easier to isolate 100,000 splenic nuclei than 100,000 haploid nuclei from testis while establishing the protocol.

22 22| Haploid sample sorting – Sort the target number of haploid nuclei – typically 100,000 in our experiments – into the previously prepared 5 mL polypropylene tube or 96 well plate. · The time required to isolate 100,000 cells is dependent on the concentration of nuclei in solution, but typically splenic samples can take as little as 2 minutes. The haploid samples tend to require 5-15 minutes to isolate 100,000 cells. Post-FACS:

23 23| Centrifuge the samples at 500 x g for 5 min at 4°C. Remove the supernatant, leaving approximately 50 µL. Do not allow the pellet to dry. At this point the nuclei can be used for a variety of applications, including single- and bulk-nuclei sequencing. The specific application will determine what buffer the nuclei should be exchanged into. Below we provide a case study where we isolated haploid sperm nuclei for library preparation with the droplet-based 10x Genomics single-nucleus ATAC-seq kit. Case Study: 10x single-nucleus ATAC-seq

Citation: Steve Novakovic, Vanessa Tsui, Tim Semple, Luciano G Martelotto, Davis J Mccarthy, Wayne Crismani SSNIP-seq: A simple and rapid method for isolation of single-sperm nucleic acid for high-throughput sequencing https://dx.doi.org/10.17504/protocols.io.6qpvr67jbvmk/v1

This is an open access protocol distributed under the terms of the **Creative Commons Attribution License**

24 24| Add 300 µL NWRB with 0.1% BSA and 0.01% Digitonin and incubate on ice for 5 minutes.

25 25| Centrifuge at 500 x g for 5 min at 4°C. Remove the supernatant, leaving behind ~50 µL. Do not dry the pellet.

26 26| Add 300 µL Dilute Nuclei Buffer (10x Genomics) and gently resuspend the nuclei by pipetting up and down 3 times using a wide-bore pipette tip.

27 27| Centrifuge at 500 x g for 5 min at 4°C. Remove the supernatant, leaving behind ~50 µL. Do not allow the pellet to dry.

28 28| Gently resuspend the nuclei by pipetting up and down 3 times using a wide-bore pipette tip.

29 29| Quantify the concentration of the nuclei suspension using a hemocytometer. We use a Leica Thunder under 40x magnification, with brightfield to locate the hemocytometer grid and fluorescence to identify the DAPI-stained nuclei. This allows users to check the integrity of the nuclei.

· For droplet-based methods of partitioning single nuclei, such as the 10x single-nucleus kits, all nuclei must be intact. · We have used this method and reliably achieved a final concentration of 400 to 450 nuclei per µL, with the aim of loading 1500 nuclei into a 10x Chromium.

30 **Library preparation**

30| Follow the manufacturer's protocol for library preparation. From here we have made high-quality "single-nucleus" and "bulk" libraries with the haploid nuclei with the Next GEM Single Cell ATAC Library Kit v1.1 (10x Genomics) and with Nextera (Illumina) DNA Library Prep Kit respectively. For bulk sequencing we aim to sequence 50,000 nuclei. Sequencing and bioinformatics considerations

31 31| The sequencing depth required per cell will vary according to experimental aims. We have found in the early stages of protocol development that a useful guide can be to sequence a given sample in two steps. First, request your sequencing platform obtain approximately 200 million paired-end reads per one thousand expected cells. Once this data has been analysed, and cell number and coverage is approximated, a second round of sequencing is performed to obtain the required depth per cell.

Citation: Steve Novakovic, Vanessa Tsui, Tim Semple, Luciano G Martelotto, Davis J Mccarthy, Wayne Crismani SSNIP-seq: A simple and rapid method for isolation of single-sperm nucleic acid for high-throughput sequencing
https://dx.doi.org/10.17504/protocols.io.6qpvr67jbvmk/v1

This is an open access protocol distributed under the terms of the **Creative Commons Attribution License**

· If the recovery of total number of nuclei recovered is lower than expected, in the first instance it is important to independently assess how many cells are present by assessing the number of barcodes with > 800,000 high-quality paired-end reads that map to the target genome. From here the threshold can be adjusted based on the researchers' decisions.

· Next time the entire experiment is performed, the number of nuclei used for library preparation can be doubled as nuclei concentration should not be limiting.

· When using bulk- or single-nucleus ATAC-seq, the nuclei permeabilization step with digitonin, or similar detergents, and the associated buffers can be re-optimised.

Citation: Steve Novakovic, Vanessa Tsui, Tim Semple, Luciano G Martelotto, Davis J Mccarthy, Wayne Crismani SSNIP-seq: A simple and rapid method for isolation of single-sperm nucleic acid for high-throughput sequencing https://dx.doi.org/10.17504/protocols.io.6qpvr67jbvmk/v1

This is an open access protocol distributed under the terms of the **Creative Commons Attribution License**
