# Supplementary material for: SSNIP-seq: A simple and rapid method for isolation of single-sperm nucleic acid for high-throughput sequencing
Source: PLoS One. 2022 Sep 29;17(9):e0275168. doi: 10.1371/journal.pone.0275168 (PMC9521801; doi:10.1371/journal.pone.0275168)
Supplement: Attachment — Submitted filename: Response to Reviewers.docx [file pone.0275168.s004.docx]

Dear Dr Drevet

We thank you and the reviewers for your thoughtful reading of our manuscript and your helpful comments. The reviewers are complimentary on the quality and value of the protocol e.g. both reviewers confirmed that the protocol “is of utility to the research community and adds value to the published literature”, and “The protocol provides a valuable addition to the field of single cell assays of testis tissue, as especially the nucleus isolation has proven tricky in the past. The description of working steps is concise, and I would feel confident following this protocol.” We address their questions, and all suggestions, below.

*Please note that references to line numbers in the manuscript are for the clean (without tracked changes) version of the revised manuscript.

**Academic Editor’s Report and Journal Requirements:**

When submitting your revision, we need you to address these additional requirements.

1. Please ensure that your manuscript meets PLOS ONE's style requirements, including those for file naming. The PLOS ONE style templates can be found at 
https://journals.plos.org/plosone/s/file?id=wjVg/PLOSOne_formatting_sample_main_body.pdf and 
<https://journals.plos.org/plosone/s/file?id=ba62/PLOSOne_formatting_sample_title_authors_affiliations.pdf>.

This has been done for both the style requirements and author affiliations.

2. We note you have not yet provided a protocols.io PDF version of your protocol and a protocols.io DOI. When you submit your revision, please provide a PDF version of your protocol as generated by protocols.io (the file will have the protocols.io logo in the upper right corner of the first page) as a Supporting Information file. The filename should be S1_file.pdf, and you should enter “S1 File” into the Description field. Any additional protocols should be numbered S2, S3, and so on. Please also follow the instructions for Supporting Information captions [https://journals.plos.org/plosone/s/supporting-information#loc-captions]. The title in the caption should read: “Step-by-step protocol, also available on protocols.io.”

This has been done. Please see S1 File attached.

Please assign your protocol a protocols.io DOI, if you have not already done so, and include the following line in the Materials and Methods section of your manuscript: “The protocol described in this peer-reviewed article is published on protocols.io (https://dx.doi.org/10.17504/protocols.io.[...]) and is included for printing purposes as S1 File.” You should also supply the DOI in the Protocols.io DOI field of the submission form when you submit your revision.

This has been done. ([dx.doi.org/10.17504/protocols.io.6qpvr67jbvmk/v1](https://dx.doi.org/10.17504/protocols.io.6qpvr67jbvmk/v1))

If you have not yet uploaded your protocol to protocols.io, you are invited to use the platform’s protocol entry service [https://www.protocols.io/we-enter-protocols] for doing so, at no charge. Through this service, the team at protocols.io will enter your protocol for you and format it in a way that takes advantage of the platform’s features. When submitting your protocol to the protocol entry service please include the customer code PLOS2022 in the Note field and indicate that your protocol is associated with a PLOS ONE Lab Protocol Submission. You should also include the title and manuscript number of your PLOS ONE submission.

This has been done.

3. Thank you for stating the following in the Acknowledgments Section of your manuscript: 
WC and DJM received funding related to this work from the Australian National Health and Medical Research Council (GNT1129757, GNT1185387).

We note that you have provided funding information that is currently declared in your Funding Statement. However, funding information should not appear in the Acknowledgments section or other areas of your manuscript. We will only publish funding information present in the Funding Statement section of the online submission form. 
Please remove any funding-related text from the manuscript and let us know how you would like to update your Funding Statement. Currently, your Funding Statement reads as follows:

 WC and DJM received funding related to this work from the Australian National Health and Medical Research Council (GNT1129757, GNT1185387).

Please include your amended statements within your cover letter; we will change the online submission form on your behalf.

This statement has been removed from the Acknowledgements.

4. Please amend either the title on the online submission form (via Edit Submission) or the title in the manuscript so that they are identical.

This has been done with the title consistent across all documents (“SSNIP-seq: A simple and rapid method for isolation of single-sperm nucleic acid for high-throughput sequencing”).

5. Please include your full ethics statement in the ‘Methods’ section of your manuscript file. In your statement, please include the full name of the IRB or ethics committee who approved or waived your study, as well as whether or not you obtained informed written or verbal consent. If consent was waived for your study, please include this information in your statement as well.

The full ethics statement is now included on lines 129-130 and reads:

“All experimental procedures were approved in writing by the St. Vincent’s Hospital Melbourne Animal Ethics Committee.”

6. Please ensure that you refer to Figure 2 in your text as, if accepted, production will need this reference to link the reader to the figure.

Figure 2 is now referenced appropriately.

**Reviewers' comments:**

**Reviewer #1:**

The paper by Novakovic et al present a method for the isolation of haploid nuclei from fresh and frozen mice testis. The methods can be applied for several purposes as indicated by the Authors, who also anticipate that the method could be used also for human specimens.
The method is of interest, however there are some points needing detailing and clarification.

Specific points:

1. The Authors do not report any data on the yield either of the global method or of the cell sorting step, whereas it appears an important detail. It appears that a huge number of haploid nuclei is lost as the method uses one testis and recoveries 100,000 (or 300,000?, line 185). Thus this method could be used for only certain applications. Have the Authors any explanation for such high cell lost? This point is important because if the method used human specimens, it is expected that the available material would be much smaller and the question is whether the starting material was sufficient with this very low yield.

We appreciate that this warrants clarification, both in the manuscript and here in this response. We have included a new Supplementary Table 1 which has data for all experiments that used this technique since it was optimised. We have also referenced a new manuscript which is on Biorxiv and under review at PNAS, which has used this technique to identify reproducible phenotypic differences between genotypes (Tsui et al, Figure 1b, https://doi.org/10.1101/2022.06.16.496499).

From a practical perspective, the protocol has been optimised for efficiency – less than an hour – and stringent quality of intact nuclei as opposed to yield. We typically set a target number of haploid cells and stop sorting once that has been reached. E.g. in a very simple example if a user wanted to use a plate-based library preparation approach, and the researcher were using a 384-well plate, they would only require 384 nuclei; one in each well. With respect to numbers such as “100,000” frequently mentioned in the original submission, this stems from aiming to obtain sequencing data for 1,000 nuclei from a droplet-based method (e.g. with 10x Genomics library preparation kits). And to prepare the sequencing libraries with an appropriate concentration of nuclei, based on constraints set by these kits and “droplet encapsulation rates”, we aim to sort 100,000 haploid cells. We have aimed to not place excessive focus on 10x kits as the intended downstream application of this method, but we appreciate that further clarification of where the target number of nuclei originates is required.

We clarify on lines 57-58;

- “We use 100,000 as a minimum target number in this protocol as droplet-based single-nucleus library preparation methods can require a high concentration of nuclei in a small volume.”

and lines 291-294.

- “For droplet-based methods of partitioning single nuclei, such as the 10x single-nucleus kits, all nuclei must be intact. We have used this method and reliably achieved a final concentration of 400 to 450 nuclei per μL, with the aim of loading 1500 nuclei into a 10x Chromium.”

From a technical perspective, using this protocol, we can routinely sort more than 300,000 haploid nuclei from a single mouse testis. However, due to our strict selection criteria for single cells (on FACS), approximately 50% of the total sorted nuclei are gated out. As our intention is to sequence individual nuclei, often with droplet-based library methods which have steps where all nuclei are in solution adjacent to one another from protocol steps 23-30 inclusive, it is essential that all nuclei included are intact. If one nucleus included is not intact and extrudes its contents into solution, it can contaminate the data of an unknown quantity of other nuclei when they are encapsulated with the “unwanted” free-floating nucleic acid. This need for the integrity of all nuclei is referred to on lines 291-292 and reads:

“For droplet-based methods of partitioning single nuclei, such as the 10x single-nucleus kits, all nuclei must be intact.”

If starting material is very low, a user can consult the newly added Supplementary Table 1 to consider this protocol could be suitable for their needs. Also, sorting individual nuclei into a plate is probably more appropriate in that situation. This comment is added to step 22 and now reads:

“If material is very limiting, it might be helpful to consider a plate-based method to maximise yield and therefore to sort one nucleus per well of an appropriate plate at this step.” [lines 261-263]

2. Cell sorting. Details on strategy to sort haploid nuclei is completely lacking. Which gate in FSC/SSC dot plot? Did you exclude doublets? Which were the technical settings of the instrumentation?

We have included a new Figure 2, which illustrates the gating strategy. The gating strategy and technical settings of the instrumentation have been expanded on in in the section “Cell sorting and cytometer settings”, from lines 198-263 and in particular in steps 19-20. This now reads:

Step 19. “Using the standard workflows of your FACS facility, gate for individual nuclei (Figure 2a-e). Gate the main population of nuclei using linear forward scatter area (FSC-A-lin) and linear side scatter area (SSC-A-lin) by region gate 1 (R1) (Fig 2A). Then gate single cells R2, R3, R4, R5 (Fig 2B-E).”

Step 20. “To discriminate haploid from diploid nuclei (R6), use the DAPI stain to quantify the DNA content of each nucleus (Fig 2F). Splenic nuclei serve as diploid controls here, which are essential to confidently gate around the peak that corresponds to the DAPI-stained haploid nuclei (Fig 2F). It is therefore important to set the voltage of the laser that excites DAPI allow visualisation of the G1 peak of the diploid sample, as well as a haploid peak, in the same range on the x-axis (Fig 2F, right). The haploid peak – on the x-axis of a histogram – will be half that of the diploid G1 peak.”

3. Line 213. The haploid peak is represented in a fluorescence distribution histogram. What do you mean with density plot? In addition, use “gate around peak” not “gate with”.

We incorrectly used the term “density plot” where we should have used “histogram”. This has been corrected, and now reads:

“The haploid peak – on the x-axis of a histogram – will be half that of the diploid G1 peak.” [lines 246-247]

We clarified the wording about gates as appropriate too. This now reads:

“Splenic nuclei serve as diploid controls here, which are essential to confidently gate around the peak that corresponds to the DAPI-stained haploid nuclei (Fig 2F)” [lines 242-244].

4. In the abstract, the Author claim that the method is reproducible, but non data on this point are provided

We have changed the word “reproducible” to “reliable”. We appreciate the word “reproducible” conjures up an expectation of “reproducible phenotypes”. Whereas we mean a “reliable protocol” that works every time, assuming other aspects of quality assurance in the user’s laboratories are met.

We have included a new Supplementary Table 1 which has data for all experiments that used this technique since it was optimised.

Minor:
Figure 1 is confused with Figure 2

We now refer, accurately, to Figure 2 [line 210].

Referring to real Figure 1 is lacking in the text

We now refer to Figure 1 in a number of appropriate sections [lines 57, 66, 133, 165].

Line 195, G1 peak does not seem centred on x-axis.

We modified the text to instruct that the G1 peak needs to be “in range”. This sentence on now reads;

“This G1 peak – of the diploid sample – is placed in-range, visually, on the x-axis by adjusting the voltage.” [lines 210-211]

**Reviewer #2:**

The protocol provides a valuable addition to the field of single cell assays of testis tissue, as especially the nucleus isolation has proven tricky in the past. The description of working steps is concise, and I would feel confident following this protocol.
However, I do have some concerns with the manuscript:

1) The authors refer to the method as “single-sperm nucleus isolation”. I find this misleading. Mouse testis contains haploid spermatids at different stages in development and transcription profiles differ distinguishably from round spermatids to later spermatid stages. The nucleus shown in figure 2C corresponds to a later spermatid stage, however FACS sorting of the haploid population would in my opinion also include more immature round spermatids. If this population is excluded in the protocol, it is unclear how. This should be discussed and Figure 2 should be updated to depict not only a single nucleus, but a representative sample of the obtained haploid nuclei.

We have modified the text and a new Figure 3 to illustrate that this protocol is designed to isolate all types of haploid cells from a male, that are available from the given tissue; from round spermatids to mature sperm. We also make clear in the text that the protocol is designed to isolation both spermatids and spermatozoa:

“This method is developed to isolate nuclei from both spermatids and spermatozoa.” [lines 61-62]

2) In line with my first concern, the authors imply that this protocol can be adapted to isolate mature human sperm. Maturation of sperm, including development of fertilisation capability and motility, is completed not in the testis, but in the epididymides. In my experience, motile sperm is “more sticky” than spermatids and it would be interesting to see how the protocol performs with this population. At least, the distinction between spermatids, spermatozoa and mature sperm should be made clear in the manuscript and discussed in more detail.

We agree that we did not make sufficiently clear that our motivation for developing this protocol was due to our own technical challenges due to the stickiness of these cell types. Therefore, we developed a method that focuses on the isolation of intact nuclei. This is now made explicit and reads:

“A major challenge for the isolation of individual haploid cells from males for sequencing is that they stick to most pipette tips and tubes, and traditional methods for bulk sperm isolation use technically challenging density gradients. Therefore, the motivation for developing this technique is to have a simple and rapid nucleic acid preparation technique that is compatible with high-throughput sequencing technologies,…” [lines 42-46].

We also have added new data in Supplementary Table 1, showing how with epididymides we used the protocol to isolate between 320,000 to 1,100,000 haploid nuclei.

3) I was unable to assess the data from the case study, as it was not made available to reviewers prior to publication. Thus, I feel a bit uncomfortable to classify this as a validated method. At least the quality control data for the case study, for example an image of isolated nuclei in the hemocytometer to assess nuclear integrity, should be included.
In light of these points, I recommend the manuscript be revised.

We have included a new Supplementary Table 1 which has data for all experiments that used this technique since it was optimised. We have modified Figure 2 as suggested to show more examples of nuclear integrity. We have also referenced a new manuscript which is on Biorxiv and under review at PNAS, which has used this technique to identify reproducible phenotypic differences between genotypes

(Tsui et al, Figure 1b, https://doi.org/10.1101/2022.06.16.496499).

**Minor**

(taken from the reviewers’ field: “Is the article presented in an intelligible fashion and written in standard English?”)

Reviewer #1: Yes

Reviewer #2: No: It is a clear and concise description, I noticed only some minor typos:
line 127 – placed

Done [line 132]

lines 151/152 – squeezing the testis with pointed tweezers until the testis bursts

Done [lines 159-160]

line 156 – microcentrifuge tube

Done [lines 164-165]

line 192 – Not a functional sentence

We modified this sentence for clarity, to read;

“Diploid control material – here, the single splenic nuclei suspension – is used to identify the G1 diploid peak based on DAPI intensity, which in turn is used to identify haploid cells from reproductive tissue (Fig 2).” [lines 208-210]
